# Antiseptic Agents for Chronic Wounds: A Systematic Review

**DOI:** 10.3390/antibiotics11030350

**Published:** 2022-03-06

**Authors:** Koko Barrigah-Benissan, Jérôme Ory, Albert Sotto, Florian Salipante, Jean-Philippe Lavigne, Paul Loubet

**Affiliations:** 1 Bacterial Virulence and Chronic Infections, INSERM U1047, Department of Microbiology and Hospital Hygiene, University of Montpellier, CHU Nîmes, 30029 Nîmes, France; koko.barrigahbenissan@chu-nimes.fr (K.B.-B.); jerome.ory@chu-nimes.fr (J.O.); 2 Bacterial Virulence and Chronic Infections, INSERM U1047, Department of Infectious Diseases, University of Montpellier, CHU Nîmes, 30029 Nîmes, France; albert.sotto@chu-nimes.fr (A.S.); paul.loubet@chu-nimes.fr (P.L.); 3 Department of Biostatistics, Epidemiology, Public Health and Innovation in Methodology, University of Montpellier, CHU Nîmes, 30029 Nîmes, France; florian.salipante@chu-nimes.fr

**Keywords:** antiseptic agents, efficiency, iodine, systematic review, wound healing, wound infection

## Abstract

In many parts of the world, antiseptic agents remain non-indicated in chronic wound care. In the current context of bacterial resistance to antibiotics and the development of new-generation antiseptic agents, wound antisepsis represents an asset for the prevention of wound infection. We aimed to evaluate four common antiseptic agents in chronic wound care complete healing. The review protocol was based on the Cochrane Handbook for Systematic Reviews of Intervention and devised in accordance with the Preferred Reporting Items for Systematic Review and Meta-Analyses (PRISMA) statement guidelines. Five databases and three clinical trials registries were searched from inception to 30 June 2021 without language restrictions. We included randomised trials evaluating the efficacy of antiseptic agents in chronic wound care in adults. Interventions considered were those using antiseptics for cleansing or within a dressing. Risk of bias was assessed using the bias excel tool provided by the Bristol Academy. Evidence quality was assessed using Grading of Recommendation Assessment, Development and Evaluation (GRADE) criteria. Of 838 studies, 6 were finally included, with a total of 725 patients. The included studies assessed iodine (cadexomer or povidone iodine) (*n* = 3), polyhexanide (*n* = 2), and octenidine (*n* = 1). Limited evidence suggested a better wound healing completion with iodine compared to saline (two randomised controlled trials (RCT), 195 patients, pooled RR 1.85 (95%CI (1.27 to 2.69)), moderate-quality evidence). There was not enough evidence to suggest a difference in wound healing using octenidine or polyhexamide. None of the antiseptic agents influenced adverse event occurrence compared to saline.

## 1. Introduction

Chronic wounds are wounds failing to proceed through the normal phases of healing in an orderly and timely manner. The definition of time without complete or partial healing differs across countries, ranging from 4 weeks to 3 months [1]. The Wound Healing Society defines four types of chronic wounds: diabetic foot ulcers (DFU), vascular ulcers (containing venous and arterial ulcers), and pressure ulcers (PU) [2].

Infection is a common complication of chronic wounds. Historically, the research on wound infection control and improvement was focused on reducing the “pathogen burden”. However, quantitative consideration of microbial load is insufficient for assessing wound improvement or wound risk of infection [3,4]. Microorganisms in a chronic wound are co-aggregated together within a protective extracellular matrix, constituting a biofilm. This biofilm conformation induces a dramatically increased tolerance to host immune defences and a greater resistance to antimicrobials [5]. Biofilm delays wound healing by inducing an ineffective host inflammatory response and damaging host tissues [6]. For this reason, a management of the biofilm is more relevant for the treatment of chronic wound [7]. In 2011, Dissemond et al. classified wounds into four categories, depending on wound bed clinical and microbiological situation: (1) contaminated or colonised wounds without risk of infection; (2) colonised wounds at risk (WAR) or critically colonised wounds; (3) wounds with local infection; and (4) systemic infections and infected wounds. The authors suggested the use of antiseptic agents for wounds from the second category alongside other treatments [8].

Chronic wound care commences with wound bed preparation via (i) wound cleansing to create a wet or moist environment, favourable to healing. (ii) Wound debridement via removal of devitalised, contaminated tissue from within or adjacent to a wound, until surrounding healthy tissue is exposed [9,10]. Debridement can be mechanical (sharp debridement, surgical), enzymatical, or bio-surgical (e.g., maggot therapy) [11,12,13,14,15]. Negative pressure wound therapy has also been used for bacterial decontamination and wound bed preparation [16]. (iii) Application of a suitable dressing, according to the type of wound. (iv) Antibiotic treatment, exclusively for infected wounds. Other therapies are beneficial to specific wounds: compression therapy is required for venous leg ulcers (VLU) [17]; arterial revascularisation, offloading foot ulcers, and diabetes control are essential in DFU [18,19]; and skin assessment and care, offloading and pressure redistribution, dressings [19,20], and structured educational program are useful for all types of chronic wounds.

In some countries, tap water or saline remain the only recommended agents for wound cleansing. Antisepsis is a common, yet controversial, wound cleansing method. Some studies consider debridement alone insufficient to reduce the biofilm that delays wound healing and suggest antiseptics to delay biofilm reformation and reduce the risk of infection [7,20]. Antiseptic agents may complement the debridement process and control infection.

The primary mode of action of antiseptics can be pharmacological, metabolic, and/or immunological [21]. For the purpose of this review, antiseptic agents are defined as medication that can prevent the growth or destroy microorganisms in or on a living tissue. Following this definition of medication, antiseptic agents must pass through a drug authorisation procedure with a medicine agency [22]. The main antiseptic agents used in chronic wound care are halogenated compounds, alcohol-based agents, biguanides (e.g., polyhexanide also called polyhexamethylenebiguanide or PHMB, chlorhexidine), and quaternary ammoniums (e.g., octenidine). Halogenated compounds include subfamilies such as the iodine/iodophor agents (e.g., povidone iodine, cadexomer iodine) and chlorous agents (hypochlorite, hypochlorous acid) [21,22]. Alternative therapeutics (e.g., honey, silver), while been antimicrobial agents are not antiseptic agents as they did not go through an authorisation procedure for this purpose [23]. They therefore are not part of the antiseptic agents’ classification. International guidelines recommend against the routine use of topical antiseptics to manage infected chronic wounds [24,25,26,27].

The emergence and diffusion of multidrug resistant bacteria and a better formulation of antiseptics with less side effects has renewed consideration of antisepsis. No resistance or adaptation has yet been observed in antiseptic agents with unspecific effects such as iodine agents, polyhexanide, octenidine, or oxidising agents (e.g., hypochlorous acid) [21]. In countries where antiseptic agents are a part of chronic wound care management protocol, there is no consensus on the best antiseptic agents for chronic wound care. Our study aimed to assess the evidence of four common antiseptic agents on chronic wound healing.

## 2. Results

### 2.1. Characteristics of the Included Studies

**Figure 1:**
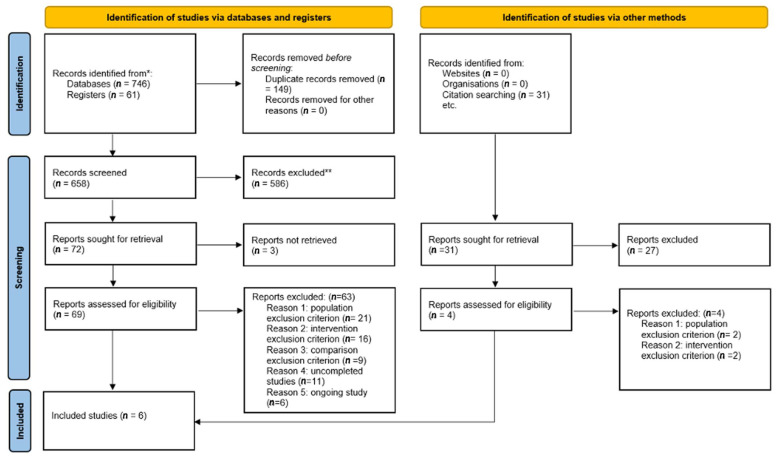
Study flow diagram.

We identified 838 studies including 61 registered clinical trials through database searching and 31 through manual searching. Of these studies, 149 duplicates were removed, and a further 613 studies were excluded by title or abstract. Three studies could not be retrieved and they were withdrawn from the database. We fully screened 73 studies, leaving 6 studies eligible for inclusion (Figure 1) [28,29,30,31,32,33]. A total of 17 studies were excluded after full screening: 23 studies met a population exclusion criterion (10 studies were not clinical trials and 13 studies included non-chronic or infected wounds); 21 out of 23 were identified through databases and registers searching and the last 2 were identified via other methods. A total of 18 studies met an intervention exclusion criterion (non-antiseptic agents or unbalanced methodology; 16 were identified by databases or register searching and the last 2 by other methods). Nine studies met a comparison exclusion criterion (no control group), 11 studies were uncompleted or prematurely ended, and six studies were ongoing. The six included studies were published from 1989 to 2020 and included 725 patients [28,29,30,31,32,33]. No unpublished studies were included.

The included studies are listed in Table S1. No studies assessing chlorous agents met our eligibility criteria. Saline was the comparator in all studies. None of the studies compared two or more antiseptic agents. Three studies evaluated the efficacy of iodine (cadexomer iodine or polyvidone iodine) [28,31,32], two studies evaluated polyhexanide [30,33], and one last study evaluated octenidine [29]. Five studies had two comparative groups of patients, and one had three comparative groups [32]. All studies were multicentre, taking place in 55 centres, both inpatient and outpatient settings across South Korea, India, France, Hungary, the UK, the USA, Germany, Canada, and Italy. Different types of chronic wounds were studied: one including DFU only [28]; one including VLU only [29]; two including PU only [30,31]; one including DFU, VLU, and PU [32]; and the last including chronic wounds without precision of the type of the wound [33]. The minimum duration used to define chronic wounds ranged from 4 weeks [28,29] to 3 months [31], although others did not state the definition, but gave the mean duration of the wounds [32], or merely described wounds as “chronic” [33], or only gave the duration of the comorbidity [30]. Study duration ranged from 4 weeks [30,33] to 24 weeks [31].

The seven domains of risk of bias were assessed following the Cochrane recommendation of 2011 [34] and are presented in Table S2. The most common source of bias was outcome assessment blinding (see Table S2). The Risk of Bias tool (2019) released by the Cochrane collaboration was also used for the risk of bias assessment and presented in Table S3. The overall risk of bias among included studies was rated with some concerns for five studies [28,29,30,32,33] and high for one [31] (Table S3). A plot of the percentage of risk of bias assessments per domain is presented in Figure S1, as recommended by the Cochrane collaboration tool for risk of bias assessment in randomised trials (2019 version).

Three studies reported the primary outcome (wound healing) as the proportion of patients with complete wound healing at 4 and 8 weeks [29], at 12 weeks [32], and as healing time from wound size [29]. All studies assessed wound healing rate as wound size reduction by planimetry measurement. Three studies assessed pain as a secondary outcome with various pain scales (e.g., verbal rating scales, visual analogue scales) [30,31,33]. Two studies evaluated bacterial bioburden reduction [31,33]. Four studies reported adverse events (AEs) [28,29,31,32]. No AEs were noted in the last two studies [30,33]. Table 1 reports the summary of findings for the primary outcome and Table S4 the different outcomes reported among the studies.

### 2.2. Iodine vs. Saline (3 Randomised Controlled Trials (RCT), 260 Patients)

Three studies, with a total of 260 patients, compared iodine to saline [28,31,32]. In 1984, Holloway et al. studied the efficacy of cadexomer iodine in 3 months venous stasis ulcers over 24 weeks [31]. The study was rated at high risk of bias. In 2018, Raju et al. also studied the efficacy of cadexomer iodine in the treatment of various 1 month chronic ulcers over 12 weeks [32]. In 2019, Gwak et al. studied the efficacy of an 8 weeks treatment of povidone iodine in patients with DFU with a mean duration of 7 weeks [28]. The risk of bias was unclear for these studies.

Two studies assessed the primary outcome as the proportion of patients with complete wound healing (Table S1) [28,32]. Gwak et al. observed no significant difference in the two groups at 8 weeks (44.4% vs. 44.1%; *p* = 0.978) [28], whereas Raju et al. found a significant complete wound healing for patients treated with two different formulations of cadexomer iodine compared to saline at 12 weeks (61.9% vs. 20%; *p* < 0.001) [32]. The pooled data showed that iodine has a higher percentage of patients achieving complete wound healing (RR 1.85 (95%CI (1.27 to 2.69), *n* = 2 studies, moderate quality evidence) (Table 2). In the last study, the endpoint was the time to complete healing (Table S1) [31]. The authors found no significant difference between the two groups (31.0 days ± 14.1 for povidone iodine vs. 33.3 ± 12.6 for saline; *p* = 6.54).

Among the three studies, two reported the healing rate (Table 2) [31,32]. Raju et al. presented the healing rate as reduction percentage in ulcer size from baseline to 12 weeks. They observed a significant reduction on wounds treated with both formulations of iodine (94.3% and 90.4%) compared to saline (67.8%) (*p* < 0.001) [32]. Holloway et al. defined the healing rate as the ulcer size reduction per week per baseline size. They noted no significant difference between patients treated with cadexomer iodine versus saline (0.04 cm^2^/week/cm^2^ ±0.01 vs. 0.03 ± 0.01, respectively; *p* = 0.079) [31]. Finally, Gwak et al. reported three indirect markers of healing rate: the percentage rate of change of wound length, width, and area [28]. They found no difference between the two treatments. The heterogeneity of the data prevented pooling. The quality of the evidence was very low.

Pain was only evaluated in one study [31]. No statistical difference was observed between the two treatments (cadexomer iodine versus saline) (*p* = 0.96) (Table 2). The quality of the evidence was low.

Finally, no studies evaluated the bacterial bioburden.

All three studies reported AEs [28,31,32]. The pooled data showed no significant difference between AE incidence in the iodine group compared to the saline group (RR 1.44 (95%CI (0.77 to 2.68), *n* = 3 studies, moderate quality evidence) (Table 2).

### 2.3. Polyhexanide Compared to Saline (2 RCT, 334 Patients)

Two studies, with a total of 334 patients, compared polyhexanide to saline [30,33], both with unclear risk of bias. In 2011, Sibbald et al. studied the efficacy of polyhexanide solution in patients with chronic wounds over 4 weeks [33]. In 2016, Bellingeri et al. studied the efficacy of polyhexanide solution in patients with PUs or mixed aetiologies of chronic ulcers, over 4 weeks [30]. Neither study reported wound healing, and thus the primary outcome could not be evaluated.

The healing rate is described in Table 3 [30,33]. Bellingeri et al. evaluated healing rate as wound improvement on the 13 item BWAT scale (Bates Jensen Wound Assessment Tool) [30]. They observed a significantly improved healing rate in the polyhexanide group (*p* = 0.072), and a significantly better score at 4 weeks compared to the first week in the experimental group (*p* = 0.025). Sibbald et al. calculated the percentage decrease of the wound surface area by planimetry measurement and comparison [33]. They noted no significant difference between the median reduction of the wound surface in the polyhexanide group versus the saline group (35% vs. 28%; *p* = 0.85). Due to the heterogeneity of the measurement tools, we could not pool the data. The quality of the evidence was low.

Concerning pain, Bellingeri et al. observed no significant difference between groups [30], whereas Sibbald et al. reported a significant pain reduction in the polyhexanide group compared to the saline group (73.1% vs. 38.1%, *p* = 0.02) [33]. We graded the quality of the evidence low.

Finally, Sibbald et al. assessed the reduction of the bacterial bioburden [33]. They noted polymicrobial microorganisms in 5.3% of the polyhexanide group wounds versus 33% in the control group (*p* = 0.016).

Bellingeri et al. reported no AEs in either group [30], but Sibbald et al. recorded two AEs as infection in the saline control group [33]. The pooled data showed a significant difference between the incidence of AEs in the polyhexanide group compared to the saline group (RR 0.2 (95%CI (0.01 to 4.18), *n* = 2 studies, low-quality evidence) (Table 3).

### 2.4. Octenidine Compared to Saline (1 RCT, 126 Patients)

One study, with 126 patients, compared octenidine to saline [29]. Vanscheidt et al. assessed the efficacy of octenidine in patients with locally infected chronic VLU that was at least 1 month old and who had had no previous or concomitant drug therapy for 12 weeks. Their study was categorised at unclear risk of bias.

Wound healing was assessed as time to complete wound closure and the proportion of patients with complete wound closure (Table S1). The time to complete wound healing was not significantly different between the groups (92 days for octenidine vs. 87 days for saline; *p* = 0.952) (Table 4). Accordingly, the proportion of patients with complete wound healing was similar (30.6% vs. 32%; *p* = 0.882). Interestingly, Vanscheidt et al. reported a significant proportion of healing for patients with ulcers larger than 6 cm^2^ and older than 6 months in the octenidine group versus the saline group (33.3% vs. 0%, respectively *p*=0.022) [29]. We graded the quality of the evidence as high.

No difference in the healing rate was noted in the octenidine group compared to the saline group (37.9% vs. 40.3%, *p* = 0.769). The octenidine group achieved better results from 5 weeks. We graded the quality of the evidence high.

The study did not assess pain or bacterial bioburden.

AEs were reported in 10 patients in the octenidine group and 19 patients in the saline group, without significant difference between the groups [29]. We graded the quality of the evidence high.

## 3. Discussion

We reviewed the RCT evidence for the use of antiseptic agents in chronic wound care in adult patients. Although saline is the main recommended product used in chronic wound cleansing, numerous clinical studies described the benefit of antiseptic agents in this situation [7,17,21,35,36,37]. A limited number of studies are available on the efficacy of antiseptic agents on chronic wound healing. More limited studies are available on the efficacy of antiseptics on pain. The trials are small, clinically heterogeneous, without clearly defined outcomes, and at high or unclear risk of bias. Of the 838 RCT identified, only 6 studies were included, representing a total of 725 patients.

Our review established a better wound healing with iodine compared to saline (2 RCT, 195 patients, RR 1.85 (1.27 to 2.69)), although the quality of the evidence was moderate. In contrast, no statistical efficacy of octenidine on healing rate (compared to saline) was seen with a high-quality evidence grade (1 RCT, 126 patients RR 1.03 (0.56 to 1.90)). Interestingly, none of the antiseptic agents influenced AE occurrence compared to saline. Notably, over half of the clinical trials have never been published. Most studies had unclear risk of bias, as previously described [38,39].

Of the 838 studies, most of them did not evaluate clinical signs of infection, and mainly focused on bacterial load reduction, a measure long deemed unsuitable [2,3,4]. Furthermore, the six included studies ignored the effect of biofilm in delaying the healing process. Of the two studies assessing microbiological impact on infection, none assessed biofilm reduction [30,31,33]. For future research, we suggest the use of dynamic models mimicking the wound environment instead of the traditional quantitative microbial load in in vitro studies [40]. This includes non-static models and the consideration of multispecies biofilm reduction over a clinically relevant time (>1 month).

Although most guidelines recommend against the use of antiseptic agents [2,4,25,27], a recent consensus suggested using hypochlorite and polyhexanide in chronic wound care [21]. We found no study demonstrating a significant effect of hypochlorite on the healing of chronic wounds. We could not assess the efficacy of polyhexanide due to the heterogeneity of outcomes between studies. However, this consensus included other types of non-healing wounds such as post-surgical or burn wounds and made no distinction between WAR score categories (colonised and infected wounds). Finally, it also included non-randomised trials, which provide lower evidence than RCTs and different systematic bias are encountered [41]. The main limitation of this guideline is the extrapolation of recommendations from various studied wounds to specific chronic wound care. Another key problem is the attribution of effect to antiseptic agents when antibiotics were systematically used in case of infected wounds.

Following the diverse interpretations of study results in recommendations, future investigations in primary research must focus on value to patients and healthcare professionals, particularly treatment choice. The design of future trials should be driven by high-priority questions. Moreover, good practice guidelines must be followed at each step (e.g., design, implementation, reporting). Assessment of complete wound healing instead of wound healing rate would be more relevant, and time to complete wound healing should be reported as the main endpoint. Future research should be controlled at least against saline, and preferably with another or multiple other antiseptic agents. Another fruitful area of research would be the impact of antiseptic agents according to wound size. Two of our included studies on two different antiseptic agents reported increased healing rate for wounds larger than 6 cm^2^ versus smaller wounds [29,31]. Further good quality evidence studies may aid decision making about the use of topical antiseptics in the management of chronic wounds.

The main limitation of this review was the great heterogeneity in study designs, methodologies, and outcomes. Overall, the six studies included in this review were too heterogeneous for pooling. We could not compare the four antiseptic agents with one another. Sensitivity analysis could not be performed due to the low number of results nor could subgroup analysis by age or type of chronic wound.

Despite its limitations, this review assesses the quality of the available data on the four selected antiseptic agents on chronic wound healing, using a well-known and robust methodology. It focuses the topic on chronic wound, reducing the bias of specific cares required for the other types of wounds.

The relative effects of topical antiseptic treatment on chronic wounds are unclear. There is insufficient evidence to determine the superiority of one antiseptic agent over the others. We could not assess the effect of hypochlorous agents on chronic wound healing. Moderate evidence suggests an improvement of wound healing with iodine compared to saline. Currently, there is not enough evidence to recommend one antiseptic agent over another in this clinical situation.

## 4. Materials and Methods

### 4.1. Selection Criteria and Search Strategy

The study is registered at PROSPERO (CRD42020213494).

The review protocol was based on the Cochrane Handbook for Systematic Reviews of Intervention (version 6.2) [42] and devised in accordance with the Preferred Reporting Items for Systematic Review and Meta-Analyses (PRISMA) statement guidelines [43]. A meta-analysis was initially planned. Due to heterogeneity in included studies, a systematic review with a summary of effect estimates was performed.

Published and unpublished RCT were eligible. Two reviewers (K.B.B., J.O.) independently screened titles and abstracts to determine eligibility and assessed the full text of retained articles. Disagreements were solved by discussion or arbitration by three independent reviewers (A.S., P.L., J.P.L.). Exclusion reasons were recorded. This study followed this PICO strategy:

1. **Population**: Our population was adult patients (≥18 years) with chronic wounds as previously defined [2]. We included studies from primary, secondary, and tertiary clinical settings. We included different types of chronic wounds (leg ulcer, DFU, PU, eschar). We excluded studies containing patients with wounds requiring specific care (acute wounds, burn wounds, systemic infected wound, postsurgical wounds).
2. **Interventions** of interest were those including antiseptics as cleansing method or within a dressing with at least weekly application.
3. **Comparative** regimens included saline solution or another antiseptic. We anticipated that interventions would consist of povidone-iodine, hypochlorite or hypochlorous acid, iodine, polyhexanide, and octenidine in the form of creams, ointments, powders, sprays, or impregnated into dressings. We included intervention schedules applying concurrent therapies (e.g., negative pressure wound therapy) if the therapy was common across study arms. We excluded (i) interventions where the antiseptic agent was not the only systematic difference between treatment groups; (ii) physical and biological therapies with antimicrobial properties, such as heat or larval therapy; (iii) studies evaluating topical antiseptics in prevention of chronical wounds or those using antiseptics as preparation for surgical treatment of ulcers; (iv) studies evaluating non-recommended antiseptics in chronic wound care and those evaluating antiseptic agents alongside antibiotic agents.
4. **Outcome**: The primary outcome was wound healing, evaluated as the proportion of patients with complete healing during follow-up and/or the time to complete wound healing (analysed using survival, time-to-event approaches). An adjustment for relevant covariates such as baseline wound area or duration were ideally used to evaluate the outcome. The secondary outcomes were healing rate (described as changes or rate of change in wound size, with adjustment for baseline size); mean pain scores; bacterial bioburden reduction; and AEs, including infection.

We searched PubMed (NLM database), MEDLINE (OvidSP), Web of Science (Thomson Reuters), Google Scholar, and Cochrane library databases, as well as 3 clinical trial registries (ClinicalTrials.gov (www.clinicaltrials.gov, accessed on 17 January 2022)), EU Clinical Trials Register (https://www.clinicaltrialsregister.eu, accessed on 17 January 2022), and World Health Organisation (WHO) International Clinical Trial Registry Platform (https://apps.who.int/trialsearch/, accessed on 17 January 2022) up to 30 June 2021 without restrictions for language, study status, date of publication, or country, using a MeSH terms string chain (Figure 1). Furthermore, we searched the reference lists of reviewed studies for relevant studies. The search strategy for all databases is presented in Appendix A.1.

### 4.2. Data Collection and Analysis

One reviewer (K.B.B.) performed data extraction and quality assessment for the included studies, validated by a second author (J.O.). Disagreements were resolved by arbitration by three independent review authors (A.S., P.L., J.P.L.). We contacted study authors for additional data if necessary. We performed data extraction using a standardised sheet, as recommended by the Cochrane Collaboration’s handbook for systematic review (trial authors, year of publication, patient population characteristics, duration of follow-up, trial design, measured outcomes, including assessment methods, objectives, results, country where trials were performed, number of participants randomly assigned to each treatment group, clinical setting, detail of interventions in each group, details of comparators in each group, source of funding, number of withdrawals, outcomes). Data are presented in Table S2.

### 4.3. Risk of Bias and Certainty of the Evidence

Two reviewers (K.B.B., J.O.) independently assessed the risk of bias of eligible studies; any disagreements were resolved by arbitration by three independent review authors (A.S., P.L., J.P.L.). Risk of bias was assessed using the bias excel tool (RoB 2 checklist, 2019) [44] as recommended by the Cochrane Handbook for Systematic Reviews of Intervention (version 6.2) [42]. The overall bias risk was rated as low, moderate, high, or unclear (also some concerns). The 7 domains of bias were also assessed for each trial, following the recommendation of the Cochrane collaboration, 2011 [34].

The overall quality of evidence of included studies was assessed using Grading of Recommendations Assessment, Development and Evaluation (GRADE) as recommended by the Cochrane Handbook for Systematic Reviews of Intervention (version 6.2) [44]. Summary tables for each antiseptic were produced with GRADEPro software considering five outcomes: complete wound healing (healing rate or proportion of patients with complete wound healing), rate of change in wound size, pain assessment, bacterial bioburden reduction, and AEs. We calculated the risk ratio (RR) for dichotomous outcomes (wound healing, AEs, infection) with 95% confidence interval (CI).

### 4.4. Role of the Funding Source

There was no funding source for this study.

## 5. Conclusions

Iodine compounds showed a better effect on chronic wound healing compared to saline. Octenidine and polyhexanide did not show any difference in this healing compared to saline. Currently, there is not enough evidence to recommend one antiseptic over another in this clinical situation. Future clinical trials assessing antiseptic agents in chronic wounds management should pay attention to include several antiseptic agents in their trial for comparison, respecting the double-blind trial and with a well-defined study population. They should establish the main efficiency criteria as complete wound healing and not only wound size reduction. A sub-group analysis based on the size of the wound would be relevant to the matter. Finally, following good practice guidelines is mandatory in every step of trials in order to avoid the numerous biases found in the assessed studies.

## Figures and Tables

**Table 1:** Description of included studies.

Study	Country	Date	Setting	Number of Participants	Diagnosis	Main Outcome (Scale Details)	Duration of the Study	Intervention	Comparator
Gwak et al., 2020 [28]	Korea	March 2016–September 2017	Multicentre 10 sites	71	DFU ≥ 1 cm^2^ post debridement and no clinical signs of infection	Proportion of patients with complete wound healing	8 weeks	PVP-I 44.4% (16/36)	Saline 44.1% (15/35)
Raju et al., 2019 [32]	India	March 2016–March 2017	Multicentre 15 sites	124	Chronic ulcers (single VLU, DFU, PU with adequate arterial blood supply)	Proportion of patients with complete wound healing	12 weeks	Cadexomer iodine ointment: 65.9% (27/41) Cadexomer iodine powder: 58.1% (25/43)	Saline 20% (8/40)
Bellingeri et al., 2016 [30]	Italy	June 2010–December 2013	Multicentre 6 sites	289	PU less than 80 cm^2^	Wound improvement measured by BWAT scale tool	4 weeks	PHMB	Saline
Vanscheidt et al., 2011 [29]	Germany, France, Hungary, UK	November 2007–December 2009	Multicentre 15 sites	126	Chronic venous ulcer locally infected	Time to complete wound healing Proportion of patients with complete wound healing	12 weeks	Octenidine 92 days 30.6% (15/49)	Saline 87 days 32.0% (16/50)
Sibbald et al., 2011 [33]	Canada	February 2008–April 2009	Multicentre 2 sites	40	Chronic wounds > 1 cm^2^	Healing rate	4 weeks	PHMB 35% reduction in wound surface	Saline 28% reduction in wound surface
Holloway et al., 1989 [31]	USA	NG	Multicentre 3 sites	75	At least a venous stasis ulcer present for a minimum of 3 months	Healing rate	24 weeks	Cadexomer iodine 0.95 cm^2^ per week	Saline 0.41 cm^2^ per week

BWAT, Bates Jensen Wound Assessment Tool; DFU, diabetic foot ulcer; NG, not given; PHMB, polyhexamethylenebiguanide; PU, pressure ulcer; PVP-I, povidone-iodine; VLU, venous leg ulcer.

**Table 2:** Summary of findings table with comparison between iodine and saline solution in chronic wound care.

Comparison One: Iodine Compared to Normal Saline for Chronic Wound Care					
Patient or Population: Chronic Wound Care Intervention: Iodine Comparison: Normal Saline					
Outcomes	Anticipated Absolute Effects * (95% CI)		Relative Effect (95% CI)	№ of Participants (Studies)	Certainty of the Evidence (GRADE)
	Risk with Normal Saline	Risk with Iodine			
Proportion of patients with complete wound healing assessed with: visual assessment follow up: range 8 weeks to 12 weeks	307 per 1000	567 per 1000 (390 to 824)	RR 1.8478 (1.2706 to 2.6874)	195 (2 RCTs)	⨁⨁⨁◯ MODERATE ^a^
Adverse events assessed with: report follow-up: range 8 weeks to 24 weeks	115 per 1000	166 per 1000 (89 to 308)	RR 1.440 (0.774 to 2.676)	270 (3 RCTs)	⨁⨁⨁◯ MODERATE ^a^
Ulcer healing rate (healing rate) assessed with: planimetry follow-up: range 8 weeks to 24 weeks	Raju et al. [32] found a significantly (*p* < 0.001) higher percentage of reduction for both formulations of iodine (94.3% and 90.4%) compared to saline (67.8%). Holloway et al. [31] found a rate reported to baseline of 0.04 ± 0.01 cm^2^/week/cm^2^ for cadexomer iodine and 0.03 ± 0.01 cm^2^/week/cm^2^ for saline. There was no significant difference (*p* = 0.079). Gwak et al. [28] presented the healing rate with three different visual displays showing the percentage change rate for the length, the width, and the area. They found no difference.			270 (3 RCTs)	⨁◯◯◯ VERY LOW ^b^^,^^c^^,^^d^
Pain evaluation (Pain) assessed with: mean rate of change follow up: mean 24 weeks	The mean rate of change in pain scores were −2.44 ± 0.4 for cadexomer iodine and −2.47 ± 0.3 with saline with a *p* = 0.96.			(1 RCT)	⨁⨁◯◯ LOW ^d^^,^^e^
**GRADE Working Group grades of evidence** ⨁⨁⨁⨁ **High certainty**: We are very confident that the true effect lies close to that of the estimate of the effect. ⨁⨁⨁ **Moderate certainty**: We are moderately confident in the effect estimate: The true effect is likely to be close to the estimate of the effect, but there is a possibility that it is substantially different. ⨁⨁ **Low certainty**: Our confidence in the effect estimate is limited: The true effect may be substantially different from the estimate of the effect. ⨁ **Very low certainty**: We have very little confidence in the effect estimate: The true effect is likely to be substantially different from the estimate of effect.					

* The risk in the intervention group (and 95% confidence interval) is based on the assumed risk in the comparison group and the relative effect of the intervention (and 95% CI). CI: confidence interval; RR: risk ratio; RCT: randomised controlled trial. (a) Missing outcome data, around 30% of patient losses during follow-up. (b) One study with a high risk of bias due to methodology risk and both studies at risk to outcome reporting. (c) Different results in the two studies. (d) Selective outcome report. (e) Study with an overall high risk of bias.

**Table 3:** Summary of findings table with comparison between polyhexanide and saline solution in chronic wound care.

Comparison Two: Polyhexanide Compared to Saline for Chronic Wound Care					
Patient or Population: Chronic Wound Care Intervention: Polyhexanide Comparison: Saline					
Outcomes	Anticipated Absolute Effects * (95% CI)		Relative Effect (95% CI)	№ of Participants (Studies)	Certainty of the Evidence (GRADE)
	Risk with Saline	Risk with Polyhexanide			
Wound healing follow-up: mean 4 weeks Not measured	Not reported			(0 studies)	-
Adverse events assessed with: report follow-up: mean 4 days	12 per 1000	2 per 1000 (0 to 50)	RR 0.2024 (0.0098 to 4.1813)	334 (2 RCTs)	⨁⨁◯◯ LOW ^a^^,^^b^
Healing rate assessed with: planimetry follow-up: median 4 weeks	Bellingeri et al. [30] found a significantly better progression of wounds in the polyhexanide group (*p* = 0.0025) using the BWAT wound assessment scale. Sibbald et al. [33] found no significant difference (*p* = 0.85) between the two groups by comparing wound surface reduction (35% vs. 28%).			334 (2 RCTs)	⨁⨁◯◯ LOW ^a^^,^^b^
Pain assessment assessed with: Pain scales follow-up: mean 4 weeks	Bellingeri et al. [30] found similar pain scores with no significant difference in the two groups (average score = 3 with minimal or no change during follow up). Sibbald et al. [33] reported significant pain reduction in the polyhexanide group compared to the saline control group (73.1% vs. 38.1%; *p* = 0.02).			(2 RCTs)	⨁⨁◯◯ LOW ^a^^,^^b^
**GRADE Working Group grades of evidence** ⨁⨁⨁⨁ **High certainty:** We are very confident that the true effect lies close to that of the estimate of the effect. ⨁⨁⨁ **Moderate certainty:** We are moderately confident in the effect estimate: The true effect is likely to be close to the estimate of the effect, but there is a possibility that it is substantially different. ⨁⨁ **Low certainty:** Our confidence in the effect estimate is limited: The true effect may be substantially different from the estimate of the effect. ⨁ **Very low certainty:** We have very little confidence in the effect estimate: The true effect is likely to be substantially different from the estimate of effect.					

* The risk in the intervention group (and 95% confidence interval) is based on the assumed risk in the comparison group and the relative effect of the intervention (and 95% CI). CI: confidence interval; RR: risk ratio; RCT: randomised controlled trial. (a) Two studies with different results. (b) Very few participants in one study.

**Table 4:** Summary of findings table with comparison between octenidine and saline solution in chronic wound care.

Comparison Three: Octenidine Compared to Saline for Chronic Wound Care					
Patient or Population: Chronic Wound Care Intervention: Octenidine Comparison: Saline					
Outcomes	Anticipated Absolute Effects * (95% CI)		Relative Effect (95% CI)	№ of Participants (Studies)	Certainty of the Evidence (GRADE)
	Risk with Saline	Risk with Octenidine			
Wound healing assessed with: Proportion of patients with complete wound healing follow-up: mean 12 weeks	242 per 1000	250 per 1000 (136 to 461)	RR 1.0313 (0.5595 to 1.9009)	126 (1 RCT)	⨁⨁⨁⨁ HIGH
Adverse events assessed with: AE report follow-up: mean 12 weeks	317 per 1000	178 per 1000 (90 to 351)	RR 0.5614 (0.2844 to 1.1081)	120 (1 RCT)	⨁⨁⨁⨁ HIGH
Healing rate assessed with: planimetry follow-up: mean 12 weeks	No difference in the healing rate of the patients in the octenidine group, compared to patients in the saline group (37.9% vs. 40.3%; *p* = 0.769) [29].			(1 RCT)	⨁⨁⨁⨁ HIGH
Pain assessment—not measured	Not reported			-	-
**GRADE Working Group grades of evidence** ⨁⨁⨁⨁ **High certainty**: We are very confident that the true effect lies close to that of the estimate of the effect. ⨁⨁⨁ **Moderate certainty**: We are moderately confident in the effect estimate: The true effect is likely to be close to the estimate of the effect, but there is a possibility that it is substantially different. ⨁⨁ **Low certainty**: Our confidence in the effect estimate is limited: The true effect may be substantially different from the estimate of the effect. ⨁ **Very low certainty**: We have very little confidence in the effect estimate: The true effect is likely to be substantially different from the estimate of effect.					

* The risk in the intervention group (and 95% confidence interval) is based on the assumed risk in the comparison group and the relative effect of the intervention (and 95% CI). CI: confidence interval; RR: risk ratio; RCT: randomised controlled trial.

## Supplementary Materials

The following supporting information can be downloaded at https://www.mdpi.com/article/10.3390/antibiotics11030350/s1, Figure S1: Plot of the percentage of risk of bias assessments at each level of risk of bias per domain. Table S1: Summary of the included studies. Table S2: Risk of bias assessment among included studies. Table S3: Review authors’ judgements about each risk of bias item for each included study. Table S4: Different outcomes reported among the studies.

## Appendix

### Appendix A.1. Medline and Cochrane Library Search

Our search strategy basis was to focus on the four main antiseptic agents recommended or used in practice: chlorous compounds, octenidine, iodophor compounds, and polyhexanide. Four different searches were conducted then combined:

- (((((chronic wound) OR diabetic foot) OR leg ulcer) OR pressure ulcer) OR eschar) AND (((iodine) OR povidone iodine OR betadine) OR cadexomer iodine).
- (((((chronic wound) OR diabetic foot) OR leg ulcer) OR pressure ulcer) OR eschar) AND ((((hypochlorite) OR hypochlorous) OR Dakin) OR Javel).
- (((((chronic wound) OR diabetic foot) OR leg ulcer) OR pressure ulcer) OR eschar) AND ((((PHMB) OR polyhexanide) OR Polyhexamethylene biguanide) OR Polyhexamethylene).
- (((((chronic wound) OR diabetic foot) OR leg ulcer) OR pressure ulcer) OR eschar) AND (Octenidine).
