# Supplementary material for: Antiseptic Agents for Chronic Wounds: A Systematic Review
Source: Antibiotics (Basel). 2022 Mar 6;11(3):350. doi: 10.3390/antibiotics11030350 (PMC8944418; doi:10.3390/antibiotics11030350)
Supplement: Supplementary file 1 [file antibiotics-11-00350-s001.zip › antibiotics-1582167-supplementary.pdf]

Supplementary Materials

Antiseptic Agents for Chronic Wounds: A Systematic Review

Koko Barrigah-Benissan [1,†], Jérôme Ory [1,†], Albert Sotto [2], Florian Salipante [3], Jean-Philippe Lavigne [1,*] and Paul Loubet [2]

[1] Bacterial Virulence and Chronic Infections, INSERM U1047, Department of Microbiology and Hospital Hygiene, Univ Montpellier, CHU Nîmes, 30029 Nîmes, France; koko.barrigahbenissan@chu-nimes.fr (K.B.-B.); jerome.ory@chu-nimes.fr (J.O.)
[2] Bacterial Virulence and Chronic Infections, INSERM U1047, Department of Infectious Diseases, Univ Montpellier, CHU Nîmes, 30029 Nîmes, France; albert.sotto@chu-nimes.fr (A.S.); paul.loubet@chu-nimes.fr (P.L.)
[3] Department of Biostatistics, Epidemiology, Public Health and Innovation in Methodology, Univ Montpellier, CHU Nîmes, 30029 Nîmes, France; florian.salipante@chu-nimes.fr
* Correspondence: jean.philippe.lavigne@chu-nimes.fr; Tel.: +33 466683202
† These authors contributed equally to this work.

Table S1. Summary of the included studies.

Reference	Objectives	Design	Setting	Population	Number of Patients	Follow up time (weeks)	Wound care frequency	Intervention: Arm 1	Comparator Arm 2	Outcomes as reported in the studies	Outcome measurement tool	Results Arm 1 vs Arm 2	Funding
Gwak et al., 2020	Evaluate the efficacy and safety of a PVP-I foam dressing for the management of DFU	Prospective, randomised controlled, open label	Multicentre	Adult ≥ 19 with DFU ≥ 1cm² post debridement and no clinical sign of infection 36/35	71 (36/35)	8	Every 3-4 days	Saline solution cleansing + PVP-I foam dressing	Saline solution cleansing + foam dressing	(1) complete wound healing: proportion of patients with complete wound healing at week 8 (2) proportion of patients with complete wound healing at week 4 (3) proportion of patients with >50% wound healing at week 8 (4) number of days to complete healing (5) ulcer healing rate: rate of size change in ulcer (6) incidence of infection	(1) (2) (3) (4) (5) wound healing by visual inspection, wound size by wound outline measure (6) signs and symptom checklist (7) dressings count (8) spontaneous patient report, patient interview and investigator examination	(1) 44·5% with PVP-I vs 44·1% (2) 22·1% with PVP-I vs 18% (3) 69·4% vs 80%, comparable (4) 31 ±15 PVP-I vs 33·27 ±12·5 comparable (5) comparable (6) 11·1% vs 11·4% (7) 16 in both in total, comparable (8) 27·8% PVP-I vs 17·1%	Mundipharma

										(7) total and mean number of dressing changes per day (8) incidence of AEs			
Raju et al., 2019	Test clinical safety and efficacy of cadexomer iodine on Indian patients with chronic wounds	Prospective, randomised, open label	Multicentre	Adults 18 - 65 with a single DFU, VLU, or PU	40/41/43	12	Daily first 4 weeks then every 3 days	Arm 1: Standard care + cadexomer iodine ointment	Arm 2: Standard care + cadexomer iodine powder Arm 3: Standard care: cleansing with saline + debridement if required + frequent applications of saline over the nonadherent absorbent cotton and gauze wound dressing	(1) percentage of reduction in ulcer size from baseline to 12 weeks (2) ulcer healing rate: mean change in ulcer size from baseline at 12 weeks in cm² (3) proportion of ulcer with complete healing at 12 weeks (4) mean change in wound evaluation score at 12 weeks	(1)(2) ulcer area measure (3) 100% epithelialisation or skin closure without drainage (4) wound evaluation score based on 5 wound parameters (oedema, pain, exudate, erythema, pus) measured on a scale of 0 to 3	(1) 67·8% vs 94·3% vs 90·4% (2) cadexomer > standard 3·94 ± 1·44 vs 5·19 ± 2·28 vs 5·19 ± 2·28 (3) 20% vs 65·9% vs 58·1 % (4) lower scores with cadexomer iodine arms	Virchow Biotech Private Limited, Hyderabad, India

Bellingeri et al., 2016	Assess clinical efficacy of PHMB vs normal saline (NS)	Prospective, randomised, single-blind	Multicentre	Adults ≥ 18 years patients with PU < 80 cm² and Braden score >10	289 (143/146)	4	At least once a weak	Irrigation + application of a pack containing PHMB for at least 10 min	Irrigation + application of a pack containing NS for at least 10 min	(1) ulcer healing rate: inflammatory signs and wound reduction (2) pain reduction (3) AEs	(1) BWAT scale, wound size measurement with a sterile ruler (2) Visual Analogue Scale (3) AE record	(1) PHMB > NS (2) similar pain in each group (3) no AE	
Vanscheidt et al., 2012	Evaluate cytotoxic effects of octenedine	Prospective, randomised, double-blind	Multicentre	Adults ≥18 years with locally infected VLU ≥ 4 weeks and < 2 years Wound ≥ 2 and ≥ 20 cm²	126 (60/66)	12	At least once and up to 3 times a week	Arm 1 Cleansing with octenedine + compression therapy with foam dressings and elastic bandages	Arm 2 Cleansing with Ringer solution + compression therapy with foam dressings and elastic bandages	(1) complete healing time, Proportion of patients with complete wound healing (2) tolerability of the treatment (3) AEs	(1) planimetry measure and calculation (2) 5-point verbal scale from very good to worse than baseline (3) AE report documentation	(1) 92 days in arm 1 and 87 in arm 2 (2) no significant difference between the two arms (3) fewer AEs in arm 1	No information
Sibbald et al., 2011	Evaluate efficacy of PHMB foam, compared to a similar non-antimicrobial foam	Prospective, randomised, double-blind	Two-centre	Patients with chronic wound > 1 cm²	40 (20/20)	4	Change 3 times a week, evaluation every 2 weeks	Cleansing with sterile water or normal saline + PHMB foam dressing	Cleansing with sterile water or normal saline + non-antimicrobial foam dressing	(1) reduction in bacterial bioburden and healing rate (2) evaluate wound colonisation using swabs (3) pain reduction (4) AEs	(1) wound surface area measure NERDS and STONEES checklist (2) Levine technique for wound and peri-wound assessment swab	(1) wound surface reduction at week 4: 35% for PHMB and 28% for control (2) 5·3% colonisation for PHMB vs	No information

									then culture and identification<br>(3) 5-point Likert verbal descriptor scale<br>(4) AE report	35% for control<br>(3) similar pain in the two groups<br>(4) 2 AEs in control group	
Holloway et al., 1989	Evaluate the use of cadexomer iodine in the management of venous stasis ulcer	Prospective, randomised, crossover	Multicentre	Adults 31-93 years with at least a venous stasis ulcer present for a minimum of 3 months	37/38	24	Daily and 2 weeks follow-up	Irrigation with saline solution then cadexomer sprinkle + cover with a dry gauze dressing + elastic compression bandage	Wet to dry dressing with saline soaked sterile gauze pad + elastic compression bandage	(1) healing rate<br>(2) reduction in bacterial bioburden<br>(3) AEs	(1) planimetry measure<br>(2) bacteriological swab followed by analysis<br>(3) AE reports	(1) 0·95 vs 0·41 cm² per week<br>(2) no difference<br>(3) minimal AEs with cadexomer and none with the control group	No information

AE, Adverse event; BWAT, Bates Jensen Wound Assessment Tool; DFU, diabetic foot ulcer; NERDS, Non-healing, Exudate, Red friable tissue, Debris and Smell; PHMB, polyhexamethylenebiguanide; PU, pressure ulcer; PVP-I, Povidone-iodine; STONEES, Size increasing, Temperature elevation, Os, New break-down, Erythema/oedema, Exudate and Smell; VLU, venous leg ulcer.

Table S2. Risk of Bias assessment among included studies.

Study	Random sequence generation	Allocation concealment	Patient and Practitioner Blinding	Outcome assessment blinding	Incomplete outcome data	Selective data reporting	Other bias	Overall
Gwak et al.	Low risk (block generation)	Low risk (no information but unnecessary)	Low risk (none but unnecessary)	Unclear risk Some concerns (no blinding but balanced intervention and adhesion to treatment)	Low risk (full details about incomplete outcome data)	Low risk (none)	Low risk (none)	Some concerns
Raju et al.	Low risk (computer-program)	Unclear risk Some concerns (no concealment but balanced adhesion)	Low risk (none but unnecessary)	Unclear risk Some concerns (no blinding but balanced intervention and probably no effect on outcome)	Low risk (explanations about patients who discontinued the study)	Unclear risk Some concerns (some outcomes as AEs are not pre-specified)	Unclear risk Some concerns (Funding)	Some concerns
Bellingeri et al.	Low risk (computer randomisation)	Low risk (sealed envelope)	Low risk (none but unnecessary)	Low risk (different team assessments)	Low risk (none)	Low risk (analysis according to a pre specified plan)	Unclear risk (short follow up time)	Low risk
Vanscheidt et al.	Unclear (randomised with no precision)	Unclear risk (blinded setting mentioned without precision)	Low risk (blinded setting mentioned without precision but unnecessary)	Low risk (double blinded study but no precision)	Low risk (lost to follow-up could influence true value)	Low risk (pre-specified outcomes)	Unclear risk Some concerns (no information on conflict of interest)	Some concerns

Sibbald et al.	Low risk (computer program randomisation)	Low risk (sealed envelopes)	Low risk (no additional data but unnecessary)	Unclear risk Some concerns (no information)	Low risk (missing data was imputed correctly)	Low risk (predefined outcomes)	Unclear risk (short follow-up time)	Some concerns
Holloway et al.	Unclear (randomisation mentioned but no details)	High risk (No blinding and interference with treatment adhesion)	Low risk (no information but unnecessary)	Unclear risk (no evidence)	Unclear risk (no information)	Unclear risk Some concerns (no predefined outcomes)	Unclear risk Some concerns (missing information)	High risk

Table S3. Review authors' judgements about each risk of bias item for each included study.

**Study ID**	**Experimental**	**Comparator**	**Outcome**	Randomisation process	Deviations from intended interventions	Missing outcome data	Measurement of the outcome	Selection of the reported result	Overall
Raju et al, 2019	iodine	saline	wound healing	+	?	+	?	+	!
Gwak et al, 2020	iodine	saline	wound healing	+	?	?	?	?	!
Holloway et al 1989	iodine	saline	wound healing	?	–	+	–	?	–
Bellingeri et al, 2016	PHMB	saline	wound healing	+	+	+	+	+	!
Sibbald et al, 2011	PHMB	saline	wound healing	+	?	+	+	?	!
Vanscheidt et al, 2011	octenedine	saline	wound healing	+	?	+	+	?	!

– High risk of bias, ! unclear risk of bias, + low risk of bias

Table S4. Different outcomes reported among the studies.

	Gwak et al.	Raju et al.	Holloway et al.	Bellingeri et al.	Sibbald et al.	Vanscheidt et al.
**Proportion of patients with complete wound healing**	X	X	-	-	-	X
**Number of days for complete wound healing**	X	-	-	-	-	X
**Healing rate: Percentage of reduction (rate of changes)**	-	X	X	X	X	X
**Adverse effect**	X	X	X	-	-	X
**Pain evaluation**	-	-	X	X	X	-
**Reduction in bacterial bioburden**	-	-	X	-	X	-
Infection incidence	X	-	-	-	X	-
Patients' satisfaction	X	-	-	-	-	X
Number of dressing changes	X	-	-	-	-	-

In bold, outcomes used in this systematic review.

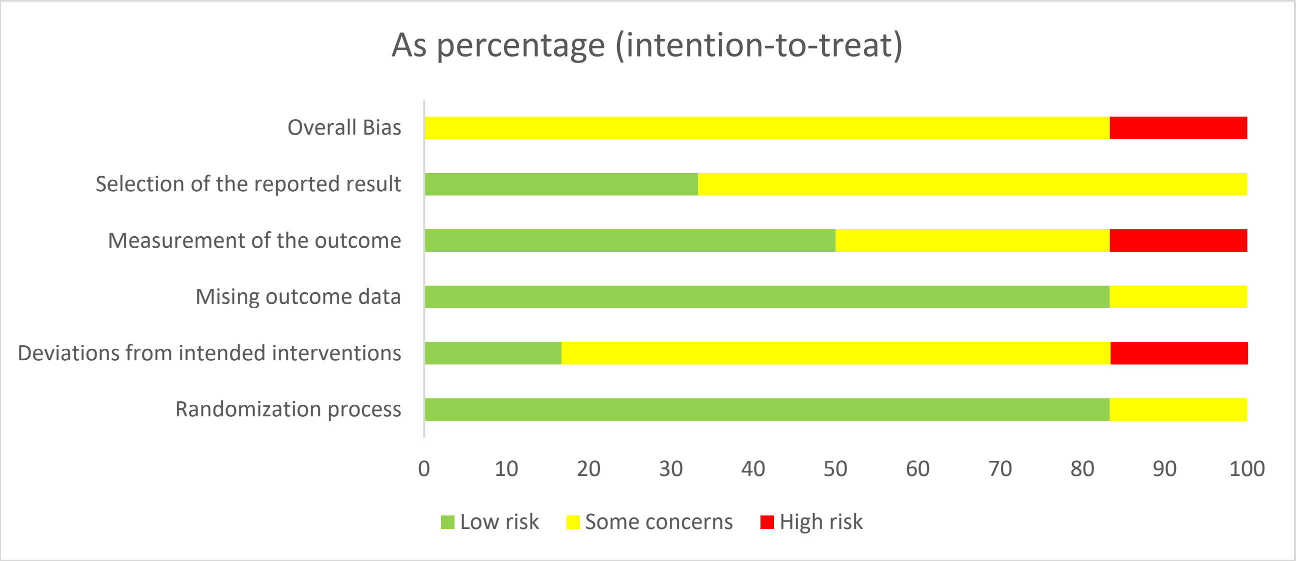

Figure S1. Plot of the percentage of risk of bias assessments at each level of risk of bias per domain.
